# Slope Aspect Modulates Alpine Meadow Spatial Homogeneity Through Niche Differences, Not Fitness Differences

**DOI:** 10.1002/ece3.74255

**Published:** 2026-09-01

**Authors:** Xiaotong Gong, Buqing Yao, Yongxiao Li, Li Ma, Renqianjie Shan, Ping Yang, Huiling Zhang, Xin Li, Yaoqing Wang, Dengshan Zhang, Huakun Zhou, Fangping Wang

**Affiliations:** ^1^ College of Ecological and Environmental Engineering Qinghai University Xining Qinghai China; ^2^ Northwest Institute of Plateau Biology Chinese Academy of Sciences Xining Qinghai China; ^3^ Key Laboratory of Adaptation and Evolution of Plateau Biota The Chinese Academy of Sciences Xining Qinghai China; ^4^ Key Laboratory of Restoration Ecology of Cold Area in Qinghai Province, Northwest Institute of Plateau Biology The Chinese Academy of Sciences Xining Qinghai China; ^5^ Qinghai Grassland Station Xining Qinghai China; ^6^ Natural Resources and Forestry and Grassland Bureau of Dari County Dari County, Golog Tibetan Autonomous Prefecture Qinghai China; ^7^ College of Life Sciences University of Chinese Academy of Sciences Beijing China; ^8^ State Key Laboratory of Plateau Ecology and Agriculture Qinghai University Xining Qinghai China

**Keywords:** fitness difference, niche difference, plant community homogeneity, Qinghai–Tibetan Plateau, slope aspect, species coexistence

## Abstract

Slope aspect modulates the spatial homogeneity of plant communities by altering species coexistence mechanisms. Modern coexistence theory posits that stable coexistence depends on the balance between niche differences (stabilizing mechanisms) and fitness differences (competitive asymmetry). How topographic factors like slope aspect modulate these mechanisms to influence community spatial patterns in alpine ecosystems remains unclear. We hypothesize that slope aspect regulates the spatial homogeneity of alpine meadow communities primarily by altering niche differences, and that this mechanism is most pronounced on environmentally stressful aspects. We surveyed the community across four slope aspects (east, south, west, north) in Maqin County, Qinghai‐Tibetan Plateau. We developed proxy indices to quantify niche difference (from phylogenetic diversity and community integrity) and fitness difference (from species evenness). Community spatial homogeneity was assessed as the inverse of spatial variability in biomass and species composition. Slope aspect significantly influenced niche differences but not fitness differences. Accordingly, homogeneity in species composition varied with aspect. Critically, variance partitioning revealed that niche difference was the primary regulator of both biomass and species composition homogeneity, but this regulatory role was significant only on the environmentally stressful west‐facing slope. Our findings demonstrate that slope aspect shapes community assembly via niche‐based filtering. The predominance of niche difference in driving spatial homogeneity specifically under the stringent conditions of west‐facing slopes underscores the context‐dependency of coexistence mechanisms and highlights the critical role of stabilizing mechanisms in maintaining community structure across topographic gradients.

## Graphical abstract

This study examines the role of slope aspect in mediating the spatial homogeneity of alpine meadow communities on the Qinghai‐Tibetan Plateau through the lens of modern coexistence theory. We found that slope aspect strongly mediated community homogeneity by altering niche differences, but not fitness differences, with this effect being significant only on the environmentally stringent west‐facing slope. These results highlight the context‐dependent nature of coexistence mechanisms, demonstrating that niche‐based assembly and stabilizing processes predominantly govern community structure across topographic gradients.

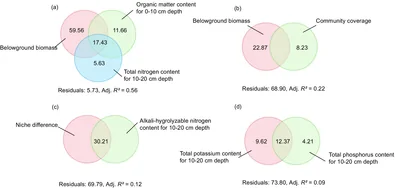

## 1 Introduction

Spatial homogenization of plant communities represents a critical challenge to ecosystems under current global change. This process not only directly leads to the loss of species richness and functional diversity, weakening community complementarity and redundancy (McKinney and Lockwood 1999), but also significantly reduces ecosystem resilience and stability in the face of environmental fluctuations and disturbances (Oliver et al. 2015). Homogenization drives communities toward simplified structures dominated by a few generalist or dominant species, thereby affecting nutrient cycling, productivity, and the sustainable provision of multiple ecosystem services. Its drivers can be categorized into environmental and anthropogenic factors (Olden et al. 2004). Topographic features, specifically slope aspect and gradient, constitute critical environmental drivers of plant community homogeneity, primarily mediated through their role in redistributing water, heat, and soil nutrients to shape heterogeneous resource patterns (Delgado‐Baquerizo et al. 2020). These features fundamentally govern the spatial patterning of plant communities across mountainous terrain, acting as primary–yet mechanistically distinct–drivers that can ultimately lead to spatial homogenization.

Slope aspect functions as a potent environmental filter that primarily drives homogenization within similar aspect categories. Through its control over solar insolation, aspect creates divergent microclimatic regimes: South‐facing slopes in the Northern Hemisphere experience intensified solar radiation, leading to higher temperatures, greater evapotranspirative demand, and ultimately drier soil conditions compared to their north‐facing counterparts (Åström et al. 2007; Wen et al. 2020). This filtering by light, temperature, and resultant moisture stress selects for a subset of species with high stress tolerance—typically drought‐adapted generalists or species with high phenotypic plasticity. Consequently, plant communities on similar aspects (e.g., all south‐facing slopes across a region) converge in composition as they are assembled from this filtered, regionally shared species pool. This within‐aspect homogenization is further reinforced by aspect‐driven soil processes. The harsher microclimate on sunny slopes can accelerate organic matter mineralization under certain conditions while reducing it under extreme aridity, and alter soil pH and nutrient availabilities (e.g., total phosphorus and nitrogen) in a consistent directional manner (Zhang, Fang, et al. 2022; Meng et al. 2020). These edaphic changes create a feedback loop that further filters root and microbial communities, strengthening the abiotic template that enforces compositional similarity. Thus, aspect acts as a large‐scale sieve, reducing niche dimensionality and promoting the dominance of abiotic stress‐tolerant species, thereby increasing spatial homogeneity among landscapes sharing the same aspect orientation (Midolo et al. 2023).

In contrast, slope gradient traditionally promotes localized heterogeneity along the topographic catena, but under specific conditions, it can also become a conduit for homogenization at broader scales. Gradient controls hydrological and geomorphic processes. Steeper slopes are characterized by rapid runoff, thinner soils, and significant downslope transport of water, sediments, and associated nutrients (Zhang, Yin, et al. 2022; Zeng et al. 2024). This creates a resource gradient from resource‐poor, erosion‐prone upper slopes to resource‐rich, depositional lower slopes. This natural redistribution fosters fine‐scale heterogeneity in soil moisture and nutrients, which can support distinct species assemblages at different slope positions (Zheng et al. 2024). However, this heterogeneity‐preserving mechanism can break down. Intensified precipitation events linked to climate change can exacerbate erosion on steep slopes to a degree that exceeds plant community resilience, stripping away soil and nutrients uniformly and leaving behind only the most ruderal, disturbance‐tolerant species across the entire gradient. Furthermore, human activities like uniform hillslope cultivation or intensive grazing disregard the natural catena, applying a homogeneous disturbance pressure that overrides the gradient's capacity to generate niche diversity. The resulting community becomes dominated by a few disturbance‐adapted generalists, leading to homogenization across the slope.

Crucially, the interplay between aspect and gradient remains poorly quantified but is likely central to predicting homogenization patterns. For instance, the erosive and homogenizing effect of a steep gradient may be amplified on a dry, south‐facing aspect with sparse vegetation cover, whereas the same gradient on a moist, north‐facing aspect with dense cover might retain more heterogeneity. Similarly, the filtering effect of a south aspect may be so severe on steep slopes that only a minimal, highly similar subset of species can persist, regardless of position on the slope. Most existing studies have simplified aspect into a binary (sunny/shady) variable and analyzed it in isolation from gradient (Zhang, Yin, et al. 2022; Xue et al. 2018), neglecting their three‐dimensional interaction. While acknowledging this potential interaction, our study focuses primarily on elucidating the dominant pathway through which aspect—a strong and consistent environmental filter—influences community assembly. We posit that the filtering effect of aspect on species pools is a first‐order driver, the mechanism of which can be deciphered through the lens of coexistence theory.

Modern coexistence theory represents species differences as the fitness difference and niche difference. In this framework, the fitness difference corresponds to equalization mechanisms that promote the competitive exclusion of species, while the niche difference corresponds to stabilization mechanisms that promote coexistence. This framework indicates that species can only coexist stably when the niche difference is greater than the fitness difference (Chesson 2000). This theory provides a general framework to explain the mechanisms of coexistence within local communities, and this framework has been widely supported by empirical evidence from biological communities. For example, the interactions between soil microbes and plants can generate the interspecies fitness difference, which can lead to the competitive exclusion of plant species when no other competitive asymmetries exist within a particular community (Wainwright et al. 2019; Yan et al. 2022). Conversely, competition can limit the evolution of niche differentiation, thus resulting in the rapid evolution of the fitness difference (Hart et al. 2019). The niche difference plays a more pivotal role in species coexistence, and this role is manifested by the fact that a reduction in niche difference can weaken the competitive advantage of species (Buche et al. 2022). Moreover, niche difference is a key factor for the successful invasion of species into existing natural communities and the primary reason for the observation of high levels of biodiversity and species coexistence (Li et al. 2019; Lyu and Alexander 2022). Quantifying the fitness difference and niche difference is necessary to apply modern coexistence theory in applied research. When Chesson (2000) first proposed this theory, he also introduced a linear quantification method based on the Lotka–Volterra model. Subsequently, this theory has been developed further to include the growth rate of invasion as a parameter in the model, including a quantitative model, invasion graphs, and methods to quantify the competitive ability (Hart et al. 2018; Spaak and Schreiber 2023). In addition, the asymmetry of competition and non‐transitive interspecies relationships has been suggested to refine the description of interspecies interactions (Ranjan et al. 2024). A sublinear model for population growth and structural methods has been used to constrain the competition coefficients for stable coexistence, thereby further improving the accuracy of the model (Hatton et al. 2024). Some studies have introduced quantitative methods from niche theory that specifically address the resource supply ratios, impact niche overlap, and demand niche overlap, thus integrating modern coexistence theory with contemporary niche theory (Letten et al. 2017). However, in natural grassland ecosystems, the scarcity of long‐term monitoring data hinders the estimation of interaction coefficients, intrinsic growth rates, and invasion growth rates of the plant community species (Hart et al. 2018). Furthermore, the methods described above tend to simplify interspecies relationships and fail to capture the composition of dozens of species in natural grasslands, thus rendering them unsuitable to assess the species coexistence patterns in large communities (Bashan et al. 2016). Quantitative methods based on community characteristics can overcome limitations in the number of species that can be considered (Keck et al. 2025). A previous study attempted to examine the mechanisms of maintenance of species coexistence using various phylogenetic indicators, but it did not account for the influence of species abundance (Liu et al. 2021).

This study replaces the estimation of fitness differences and niche differences with observable proxies for a pattern consistent with coexistence mechanisms, rather than directly estimating these indicators. Direct estimation of coexistence mechanisms requires long‐term time series data for observation, while proxies can quickly assess the key indicators of species coexistence in large‐scale multi‐species communities. Thus, this study incorporates phylogenetic diversity, community integrity, and diversity into the methods to quantify both the fitness difference and niche difference, thus jointly considering the influence of evolutionary relationships and community composition characteristics on coexistence. These proxies can capture the relative dominance patterns within the community, the results of interspecies competition, as well as the degree of differentiation of functional characteristics and phylogenetic. Although they cannot completely replace direct estimation, these proxies have been widely applied in community ecology. The phylogenetic conservatism and the theory of resource monopoly by dominant species provide sufficient rationality for these proxies. This innovative method thereby broadens the applicability of modern coexistence theory and holds promise for developing new tools to assess ecosystem conservation, as the integrated analysis of phylogenetic diversity, species richness, and evenness provides a robust means to unravel plant community homogenization processes.

Building on this theoretical and empirical background, we focus on the mechanism by which slope aspect, as a key topographic filter, influences community spatial structure through the components of modern coexistence theory. We hypothesize that in alpine meadows, slope aspect regulates the spatial homogeneity of plant communities chiefly by modulating niche differences among species, rather than fitness differences. Furthermore, we hypothesize that the role of niche difference as a stabilizing force will be most pronounced on the most environmentally stressful slope aspect (i.e., the west‐facing slope, characterized by intense afternoon radiation and relative aridity). Specifically, we address the following questions: (1) Does slope aspect differentially affect the two components of modern coexistence theory—fitness difference versus niche difference? (2) How do these components influence the spatial homogeneity of community biomass and species composition? (3) Is the regulatory role of niche difference in mediating community homogeneity strongest on the environmentally stressful west‐facing slope? By integrating a spatially explicit assessment of homogeneity with proxy indices of coexistence mechanisms, this study aims to establish a testable, mechanistic framework for understanding how topographic filtering governs community assembly in high‐altitude ecosystems through niche‐based processes.

## 2 Materials and Methods

### 2.1 Study Site

The sampling site was located near the airport in Dawu Town, Maqin County, Golog Tibetan Autonomous Prefecture, Qinghai Province, China (34°27′52″ N, 100°13′19″ E). A contour map of the study area with sampling locations is provided in Figure S1. The region has an annual average temperature between −3.8°C and 3.5°C and an annual average precipitation of 508.1 mm; with precipitation concentrated from June to September (Zhang et al. 2024). The soil composition primarily consists of alpine cold desert soil, alpine meadow soil, alpine meadow grassland soil, and leached brown soil (Liu et al. 2023). The study site is located at an elevation of 3930 m a.s.l. with no period of absolute frost‐free days throughout the year. The dominant species include *Kobresia myosuroides* , *Ligularia virgaurea*, *Poa annua* , *Festuca rubra* , *Saussurea amara* , *Oxytropis ochrocephala*, *Elymus nutans*, and *Gentiana straminea* among others. In this region, slope aspect creates strong microclimatic gradients. South‐ and west‐facing slopes receive higher cumulative solar radiation, with particularly intense afternoon sun on west‐facing slopes, leading to higher evaporative demand and generally drier soil conditions compared to north‐ and east‐facing slopes. This pattern is well‐supported by data on aboveground biomass (Table S1). Consequently, the west‐facing slope in our study system represents a more environmentally stressful habitat characterized by combined thermal and moisture stress, which serves as a key context for testing the hypothesized shift in community assembly mechanisms.

### 2.2 Community Survey and Sample Collection

**FIGURE 1:**
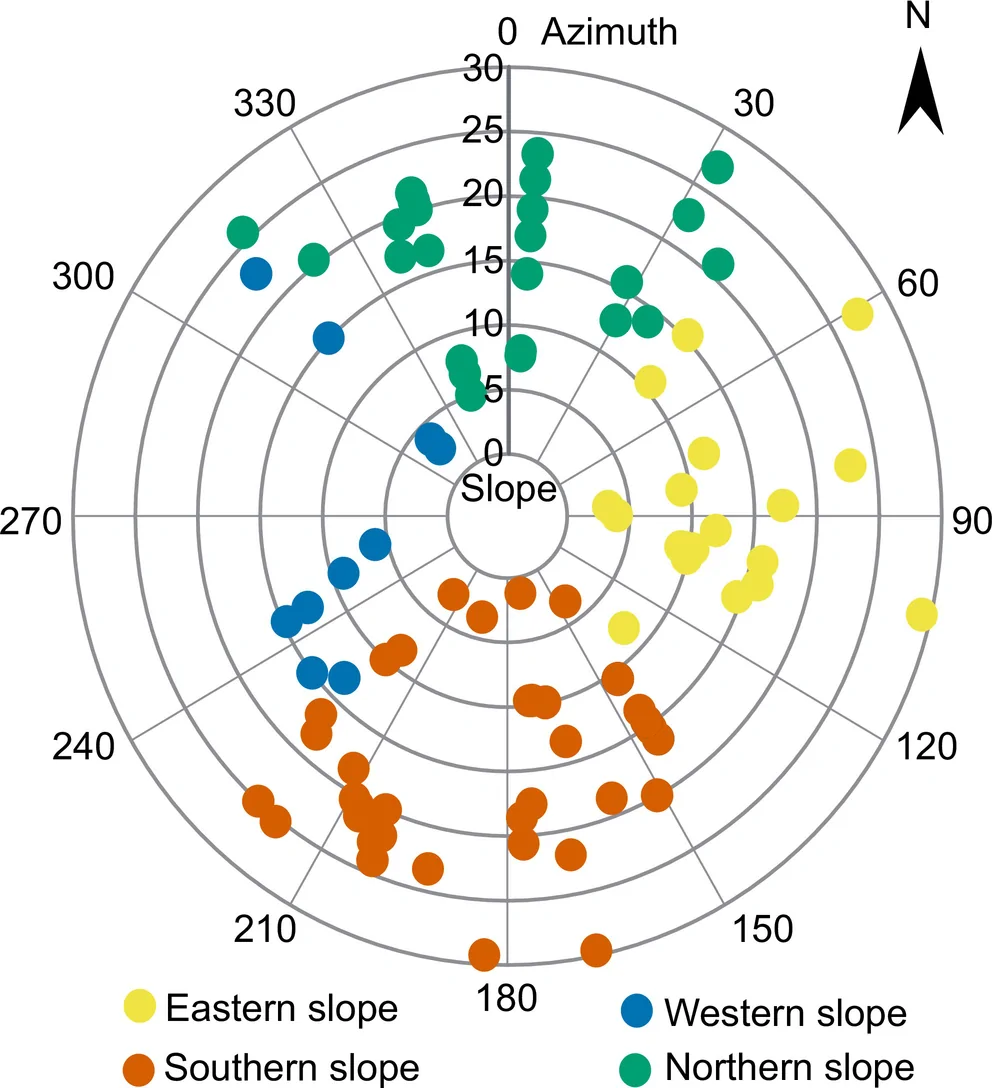
Topography of sampling points. The radial distance of each point represents the slope, while the polar angle (measured clockwise from the vertical axis) indicates the azimuth.

To isolate the effects of slope aspect and gradient from broader‐scale climate and geological variations, we selected a sampling site at a mountain top where natural gradients radiated in all directions from the summit. The northern slope of the mountain top was an alpine shrub meadow, while the southern slope was an alpine meadow. The transitional zones between the northern and southern slopes consisted of a grassland meadow. We selected four slope aspects—eastern, southern, western, and northern—based on mountain gradient and plant growth conditions. Within each aspect, we established two sample plots, and a series of quadrats were then placed within each plot for vegetation and soil sampling. All quadrats were located within a 30 m elevation range, regardless of aspect or plot. The slope aspects evaluated were defined as follows: 315° to 0° to 45° as the northern slope (including 45°), 45° to 135° as the eastern slope (including 135°), 135° to 225° as the southern slope (including 225°), and 225° to 315° as the western slope (including 315°). The slope gradients were classified according to the land management standard Code of Practice for Gradation and Classification on Grass Land (TD/T 1108–2025), with grades of 0°–5° classified as flat land, 5°–15° as a gentle slope (including 5°), 15°–25° (including 15°) as a moderate slope, and 25°–30° (including 25°) as a steep slope. Field measurements and sampling were conducted in August 2019 during the peak growing season. A moderate rainfall occurred 6 days before sampling, with no rain during the sampling period. Within each sample area, quadrats (0.5 × 0.5 m) were established at different aspects. The orientation and gradient data for each quadrat were recorded (Figure 1). We measured plant community coverage and height, and recorded the number of plant species within each quadrat as species richness. The plant aboveground parts were collected and sorted by species, oven‐dried at 65°C to a constant weight in an indoor laboratory, and weighed to obtain aboveground biomass. Within each quadrat, four replicate soil cores were collected from each of three depth intervals (0–10, 10–20, and 20–30 cm) using a soil auger, then stored in self‐sealing bags and labeled. The root and soil samples were then separated in an indoor laboratory. Belowground biomass was measured by oven‐drying roots to a constant weight at 65°C. The biomass of each soil layer was recorded. We calculated the root‐to‐shoot ratio and the proportion of belowground biomass for the 0–10, 10–20, and 20–30 cm layers relative to the total belowground biomass.

### 2.3 Measurement of the Soil Physicochemical Properties

We evaluated soil physiochemical properties for the top two soil layers (0–10 cm and 10–20 cm). Soil water content was determined by oven‐drying at 105°C for 72 h. Soil chemical properties were determined as follows: Alkali‐hydrolyzable nitrogen content was determined using the alkaline diffusion method (Khan et al. 2001). Total nitrogen content was determined using the Kjeldahl method. Organic matter content was measured using the oil‐bath heating potassium dichromate oxidation method. Total phosphorus content was measured using the alkali hydrolysis‐molybdenum‐antimony colorimetric method, and available phosphorus content was measured using the NaHCO_3_ extraction‐molybdenum‐antimony colorimetric method (Feng et al. 2023). Acid‐soluble potassium content was measured using the nitric acid extraction method (Graley 1978). Available potassium content was determined using extraction with ammonium acetate followed by flame photometry, and slow‐release potassium content was calculated as the difference between acid‐soluble potassium and available potassium. Total potassium content was measured using the alkaline fusion‐flame photometry method (Kitagawa et al. 2018). All measurements were performed for both the 0–10 cm and 10–20 cm soil layers.

### 2.4 Theoretical Rationale and Calculation of Coexistence Indices

Direct estimation of niche and fitness differences from dynamic models is impractical for species‐rich, perennial grassland communities. Therefore, we developed spatially informed proxy indices grounded in the conceptual core of modern coexistence theory (Chesson 2000).

Fitness Difference (Competitive Hierarchy): This concept reflects the degree to which one species outcompetes another under identical conditions. At the community level, a greater imbalance in species abundances (i.e., lower evenness) indicates stronger dominance and, by extension, greater fitness differences among species. Moreover, the Pielou's evenness index reflects the uniformity of species distribution within the community. Therefore, we used the inverse of the standardized Pielou's evenness index as a proxy. The equations used were: Pielou's evenness index = Shannon − Wiener diver index/ln(Species richness index), Pielou's evenness index′ = Pielou's evenness index/Pielou's evenness index_max_, and Fitness difference = 1/Pielou's evenness index′. Here, the Shannon − Wiener diversity index was calculated using the vegan package in R as ∑*p*_*i*_ × ln*p*_*i*_, where $p_{i}$ represents the relative biomass (instead of relative abundance) of species *i* in the quadrat. The Pielou's evenness index′ denotes the relative evenness index, and the Pielou's evenness index_max_ is the maximum evenness index within the community.

Niche Difference (Stabilizing Mechanisms): This component reflects mechanisms that reduce interspecies competition relative to intraspecific competition. Greater phylogenetic distance often correlates with greater functional divergence and differentiation in resource use or tolerance—key facets of niche differentiation (Zepeda and Martorell 2021). We therefore quantified niche difference as the product of community integrity (the proportion of the local species pool present, reflecting the potential for niche packing; Keck et al. 2025) and the standardized mean nearest taxon distance (reflecting the degree of phylogenetic, and by inference, functional divergence among co‐existing species; Liu et al. 2021). This multiplicative index captures both the completeness of the community and the degree of niche differentiation within it. In this study, the “species pool” is defined as the complete site‐level species pool across all aspects, in order to reflect the species integrity of the community of quadrat within the regional context. To eliminate the effect of dimensionality, we divided the mean nearest taxon distance by its observed maximum value to derive the relative mean nearest taxon distance. The equations used were: community integrity = species richness/total number of species, mean nearest taxon distance′ = mean nearest taxon distance/mean nearest taxon distance_max_, niche difference = community integrity × mean nearest taxon distance′. Here, the mean nearest taxon distance was calculated using the vegan and picante packages in R as mean nearest taxon distance = (1/species richness) × ∑mind_*ij,i*=*j*_, where $d_{\mathrm{ij}}$ is the phylogenetic distance between species *i* and *j*.

### 2.5 Calculation of the Homogeneity Indices

We calculated productivity homogeneity based on plant community biomass, defining homogeneity as the inverse of spatial variability across slope gradients (Wang and Loreau 2014). For each slope aspect, the quadrat with the lowest micro‐topographic slope gradient was selected as the baseline quadrat (assumed minimally affected by slope). A greater biomass difference between other quadrats and the baseline indicated a larger slope impact and less stable primary productivity. We used the absolute value of the denominator to eliminate the influence of relative biomass variation, followed by log transformation for standardization. A smaller homogeneity index indicated a less stable community. The formula used was: productivity homogeneity_*x*_ = ln(biomass_0_/|biomass_*x*_—biomass_0_|), *x* ≠ 0, where productivity homogeneity_*x*_ is the primary productivity homogeneity of quadrat *x* for any given aspect; biomass_0_ is the biomass of the quadrat with the lowest gradient in that aspect, and biomass_*x*_ denotes the biomass of quadrat *x*.

Species homogeneity was determined using the Jaccard similarity index. The quadrat with the lowest gradient in each aspect served as the baseline quadrat. A higher similarity index between the various quadrats and baseline plot indicated a lower slope effect on the community and thus, greater homogeneity. The formula used for the calculation was: species homogeneity_*x*_ = species richness_*x*0_/(species richness_*x*_ + species richness_0_—species richness_*x*0_), where species homogeneity_*x*_ is the species homogeneity of quadrat *x*; species richness_*x*_ represents the number of species in quadrat *x*; species richness_0_ is the number of species in the baseline quadrat, and species richness_*x*0_ is the number of species common to both quadrat *x* and the baseline quadrat. To test whether the core indicators (species homogeneity and productivity homogeneity) depend on the selection of specific baseline quadrats within each slope direction, we conducted sensitivity analyses using the Bootstrap resampling method. The results show that the productivity homogeneity is not dependent on a specific baseline quadrat (Figure S4).

### 2.6 Statistical Analysis

All statistical analyses and graph rendering were conducted using R version 4.4.2. Our analysis was designed to test hypotheses focused on slope aspect effects. Due to field constraints, the number of quadrats per aspect varied (East: 19, South: 39, West: 10, North: 24). We employed strategies to ensure robustness: First, we addressed sample size imbalance by primarily using non‐parametric tests (Scheirer‐Ray‐Hare and Kruskal–Wallis), which are valid under such conditions. We assessed differences in soil properties, coexistence indices, and homogeneity metrics across the four slope aspects using the Kruskal–Wallis test. To evaluate the main and interactive effects of slope aspect and gradient, we used the Scheirer‐Ray‐Hare test, a non‐parametric equivalent of two‐way ANOVA. These tests operate on ranks and are robust to unequal sample sizes and non‐normality. Second, for variance partitioning, we report adjusted *R* ^*2*^ values, which account for model complexity and differing sample sizes. We identified factors influencing species coexistence patterns using random forest regression (randomForest package, 500 trees), with predictor importance assessed via %IncMSE. We performed variance partitioning analysis (vegan package) to quantify individual and joint explanatory contributions of different drivers to homogeneity indicators. Figures were generated using the ggplot2 package.

## 3 Results

### 3.1 Topographic Effects on the Soil Physical and Chemical Properties

The two‐way ANOVA and Scheirer–Ray–Hare (S–R–H) test (Table 1) indicated extremely significant (*p* < 0.001) main effects of slope aspect on the contents of alkali‐hydrolyzable nitrogen, total nitrogen, organic matter, and total phosphorus in the 0–10 cm surface soil and the contents of alkali‐hydrolyzable nitrogen, total nitrogen, organic matter, total phosphorus, total potassium, acid‐soluble potassium, and slow‐release potassium in the 10–20 cm subsurface soil. The main effects of slope aspect on the contents of available phosphorus and acid‐soluble potassium in the 10–20 cm subsurface soil were highly significant (*p* < 0.01). Slope gradient had no significant main effect on these soil properties, and there was no significant interaction between aspect and gradient.

**TABLE 1:** Two‐way ANOVA and Scheirer–Ray–Hare test of the slope aspect and gradient on the physical and chemical properties of soil.

Index	Slope aspect	Slope gradient	Slope aspect × slope gradient
Soil water content	5.98	1.14	4.96
0–10 cm			
Alkali‐hydrolyzable nitrogen	16.41***	3.55	4.47
Total nitrogen	13.78***	2.02	0.54
Organic matter	35.32***	2.15	3.40
Total nitrogen	26.26***	1.70	6.67
Total potassium	8.42*	3.31	0.75
Available phosphorus	6.44	5.66	8.90
Acid‐soluble potassium	5.31	7.18	3.93
Available potassium	3.92	0.87	5.43
Slow‐release potassium	5.76	7.23	3.04
10–20 cm			
Alkali‐hydrolyzable nitrogen	41.99***	1.91	5.04
Total nitrogen	45.44***	0.85	2.84
Organic matter	48.65***	1.54	3.23
Total nitrogen	13.63***	1.36	1.54
Total potassium	28.49***	1.28	7.96
Available phosphorus	12.22**	1.38	7.93
Acid‐soluble potassium	17.78***	0.68	8.89
Available potassium	6.77	0.89	2.56
Slow‐release potassium	19.47***	1.26	9.05

*Note:* The degree of freedom for the main effect was 3, while that for the interaction effect was 7. The values in the table are *F*‐statistics and *H*‐statistics; the contents of total nitrogen and organic matter for the 0–10 cm deep soil layer and the content of acid‐soluble potassium for the 10–20 cm deep soil layer were analyzed using a two‐way ANOVA, with *F*‐statistics reported, while the remaining indicators were analyzed using the S‐R‐H test, with *H*‐statistics accordingly reported instead.

* *p <* 0.05.

** *p <* 0.01.

*** *p <* 0.001.

One‐way ANOVA and Kruskal–Wallis (K–W) tests (Table 2) showed that soil water content on the northern slope was significantly higher than on the western slope (*p* < 0.05) by approximately 29.5%, but no significant differences were observed between eastern and southern slopes. The contents of alkali‐hydrolyzable nitrogen, total nitrogen, organic matter, and total phosphorus in each soil layer were significantly higher on western and northern slopes than on eastern and southern slopes (*p* < 0.05). In the surface soil (0–10 cm), total potassium and slow‐release potassium contents were significantly higher on eastern and southern slopes than on western and northern slopes (*p* < 0.05); available phosphorus content was significantly higher on the northern slope than on eastern and southern slopes (*p* < 0.05); acid‐soluble potassium content was significantly higher on the southern slope than on western and northern slopes (*p* < 0.05); and available potassium content was significantly higher on the northern slope than on the western slope (*p* < 0.05). In the subsurface soil (10–20 cm), total potassium content was significantly higher on the southern slope than on the northern slope (*p* < 0.05); available phosphorus content was significantly higher on the northern slope than on the western slope (*p* < 0.05); acid‐soluble potassium and slow‐release potassium contents were significantly higher on eastern and southern slopes than on the northern slope; and available potassium content was significantly higher on the northern slope than on eastern and western slopes (*p* < 0.05). Except for available potassium in surface soil and available phosphorus and potassium in subsurface soil, all other soil properties differed significantly between the combined eastern‐southern slopes and the combined western‐northern slopes. Together, these data reveal a pattern of resource differentiation across aspects. Critically, the west‐facing slope exhibited a unique resource syndrome: although total nitrogen and organic matter were high (comparable to the north‐facing slope), it had the lowest values of several key available resources (soil water content, available potassium, and certain exchangeable potassium fractions). When combined with intense afternoon solar radiation, these factors collectively generate a high level of environmental stress, albeit one that is not captured by any single variable.

**TABLE 2:** One‐way ANOVA and Kruskal–Wallis (K–W) tests for the soil physics and nature of chemistry of the different slope aspects.

Index	Eastern slope	Southern slope	Western slope	Northern slope
Soil water content	21.65 ± 6.65^ab^	22.18 ± 5.71^ab^	19.34 ± 5.01^b^	25.04 ± 4.94^a^
0–10 cm				
Alkali‐hydrolyzable nitrogen	179.54 ± 31.89^b^	185.47 ± 31.21^b^	243.00 ± 52.34^a^	260.94 ± 33.52^a^
Total nitrogen	5.25 ± 1.71^b^	5.28 ± 1.71^b^	8.75 ± 0.81^a^	9.30 ± 1.31^a^
Organic matter	114.40 ± 34.75^b^	109.95 ± 25.88^b^	184.11 ± 14.89^a^	203.51 ± 31.93^a^
Total nitrogen	672.52 ± 101.74^b^	671.58 ± 68.03^b^	816.81 ± 79.46^a^	796.31 ± 77.45^a^
Total potassium	16.51 ± 0.46^a^	16.57 ± 0.35^a^	16.17 ± 0.31^b^	16.02 ± 0.77^b^
Available phosphorus	6.02 ± 3.40^b^	6.16 ± 2.88^b^	7.12 ± 3.08^ab^	9.59 ± 4.08^a^
Acid‐soluble potassium	809.25 ± 164.07^ab^	872.91 ± 92.81^a^	711.42 ± 143.55^b^	742.17 ± 143.83^b^
Available potassium	168.16 ± 57.23^ab^	175.46 ± 38.18^ab^	150.00 ± 28.91^b^	192.04 ± 47.64^a^
Slow‐release potassium	641.10 ± 149.58^a^	697.45 ± 83.80^a^	561.42 ± 153.65^b^	550.13 ± 137.08^b^
10–20 cm				
Alkali‐hydrolyzable nitrogen	172.14 ± 45.86^b^	184.88 ± 32.40^b^	214.57 ± 13.84^a^	214.89 ± 45.44^a^
Total nitrogen	4.79 ± 1.67^b^	5.26 ± 1.10^b^	6.69 ± 0.84^a^	7.06 ± 1.07^a^
Organic matter	94.52 ± 33.87^b^	104.13 ± 22.86^b^	136.57 ± 15.02^a^	150.15 ± 24.99^a^
Total nitrogen	669.06 ± 123.54^b^	683.48 ± 86.58^b^	809.15 ± 116.79^a^	817.30 ± 85.25^a^
Total potassium	17.483 ± 0.94^ab^	17.83 ± 0.88^a^	17.57 ± 0.78^ab^	17.29 ± 0.95^b^
Available phosphorus	2.03 ± 1.27^ab^	1.99 ± 1.11^ab^	1.52 ± 0.93^b^	2.22 ± 0.94^a^
Acid‐soluble potassium	755.26 ± 203.11^a^	738.36 ± 219.66^a^	693.63 ± 159.59^ab^	637.35 ± 186.89^b^
Available potassium	96.68 ± 19.13^b^	99.71 ± 17.36^ab^	93.13 ± 11.74^b^	111.70 ± 27.54^a^
Slow‐release potassium	658.58 ± 207.00^a^	638.65 ± 221.11^a^	600.51 ± 155.14^ab^	525.65 ± 192.86^b^

*Note:* Contents of total nitrogen and organic matter for the 0–10 cm deep soil layer and acid‐soluble potassium for the 10–20 cm deep soil layer were analyzed using a one‐way ANOVA, while the remaining indicators were analyzed using the K–W test. The values in the table are the mean ± SD, and the different lowercase letters indicate significant differences among the habitats of different aspects (*p* < 0.05).

To summarize the stress inference directly from measured variables: indicators supporting higher stress on the west‐facing slope include its significantly lower soil water content, surface available potassium, and exchangeable potassium fractions, as well as lower available phosphorus in the subsurface compared to the north‐facing slope. In contrast, total nitrogen and organic matter on the west‐facing slope were not lower; they were comparable to those on the north‐facing slope. Thus, the stress is not a simple overall resource impoverishment but rather a specific shortage of water and readily available potassium combined with high insolation (inferred from aspect). We explicitly note that direct microclimate measurements (e.g., temperature, evaporative demand) are not available; the stress interpretation is therefore an inference based on soil available resources and topographic context.

### 3.2 Topographic Effects on the Components of Modern Coexistence Theory

**FIGURE 2:**
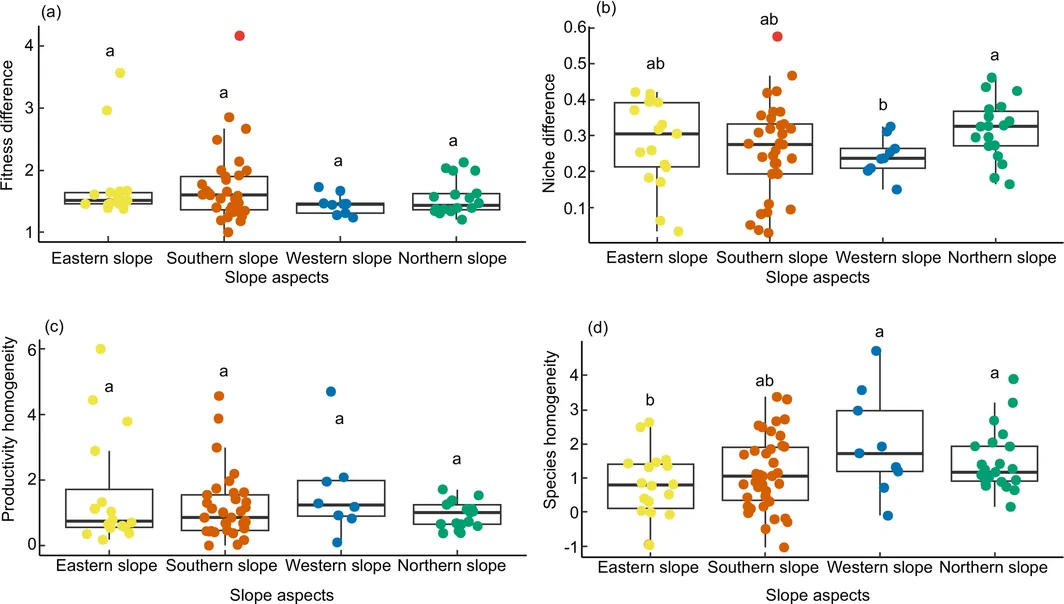
Kruskal–Wallis (K–W) test for coexistence indicators of different slope aspect and homogeneity indicators. (a) Box plot of fitness difference; (b) Box plot of niche difference; (c) Box plot of productivity homogeneity; (d) Box plot of species homogeneity. Different lowercase letters indicate significant differences among habitats of different aspects according to the K–W test (*p* < 0.05).

The S–R–H test for species coexistence indicators (Table 3) showed that aspect had no significant effect on fitness difference, but had a significant main effect on niche difference (*p* < 0.05). K–W tests (Figure 2b) indicated that niche difference on the western slope was significantly lower than on the northern slope (*p* < 0.05), while no significant differences were observed for eastern and southern slopes compared to others. For niche difference, we calculated the standardized value based on the average pairwise phylogenetic distance (MPD), multiplying community integrity by MPD. The results (Figure S2) show that the key conclusions have not undergone substantial changes. Gradient had no significant effect on either fitness difference or niche difference, and there was no significant interaction between gradient and aspect.

**TABLE 3:** Scheirer–Ray–Hare test of the slope aspect and slope gradient on the community coexistence indices and homogeneity.

Index	Slope aspect	Slope gradient	Slope aspect × slope gradient
Fitness difference	0.76	0.14	6.67
Niche difference	8.99*	0.59	4.27
Primary productivity homogeneity	3.93	4.42	2.62
Species homogeneity	11.59**	0.40	1.40

*Note:* The degree of freedom for the main effect was 3, and that for the interaction effect was 7. The values in the table are *H*‐statistics.

* *p* < 0.05.

** *p* < 0.01.

### 3.3 Topographic Effects on the Community Spatial Homogeneity

The main effect of slope aspect on productivity homogeneity was not significant, but its main effect on species homogeneity was significant (*p* < 0.01; Table 3). K–W test results indicated no significant difference in primary productivity homogeneity among aspects (Figure 2c). Species homogeneity on the eastern slope was significantly lower than on both western and northern slopes (*p* < 0.05), while species homogeneity on the southern slope did not differ significantly from others (Figure 2d). Within the altitude range studied (less than 30 m), no significant effect of gradient on productivity homogeneity and species homogeneity was detected, and there was no significant interaction between gradient and aspect.

### 3.4 Factors Affecting the Species Coexistence Patterns

**FIGURE 3:**
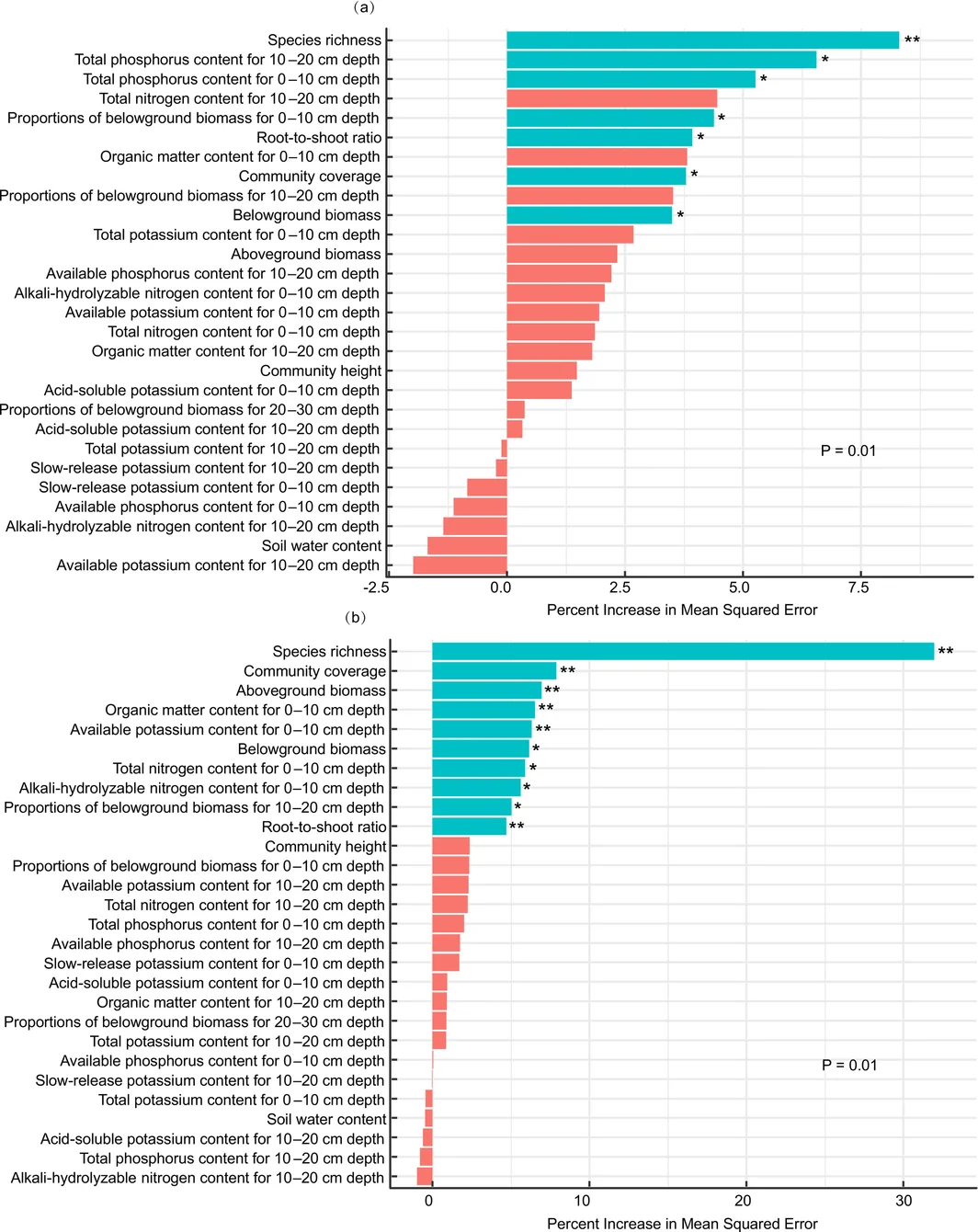
Random forest analysis of the factors affecting coexistence metrics. (a) Random forest analysis of the fitness difference. (b) Random forest analysis of niche difference. *p < 0.05. **p < 0.01.

Random forest analysis identified key factors affecting species coexistence patterns. Both community characteristics and soil nutrient contents significantly impacted fitness difference and niche difference, with more community‐related factors involved. Community characteristics influencing fitness difference included species richness, proportion of belowground biomass in the 0–10 cm layer, root‐to‐shoot ratio, community coverage, and belowground biomass; the relevant soil nutrient factor was total phosphorus content (Figure 3a). Community characteristics influencing niche difference included species richness, community coverage, aboveground biomass, belowground biomass, proportion of belowground biomass in the 10–20 cm layer, and root‐to‐shoot ratio; relevant soil nutrient factors included organic matter, available potassium, total nitrogen, and alkali‐hydrolyzable nitrogen content in the 0–10 cm layer (Figure 3b).

### 3.5 The Role of Coexistence Mechanisms in Regulating Homogeneity

**FIGURE 4:**
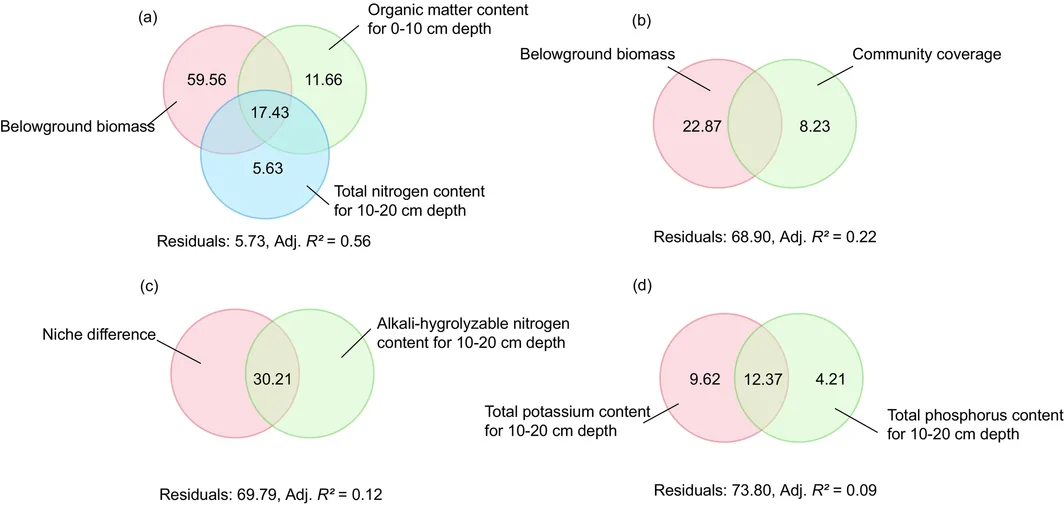
Variance partitioning analysis of productivity homogeneity. The numbers in the figure represent the standardized interpretation ratios (%) (Values < 0 not shown). (a) Eastern slope and (b) Southern slope and (c) Western slope and (d) Northern slope.

Overall, the unique contribution of niche difference to productivity homogeneity on the western slope was modest (adj. *R* ^2^ = 0.12 in Figure 4), leaving 69.79% of the variance unexplained. Nevertheless, this small but significant fraction is consistent with the hypothesis that niche difference plays a non‐negligible role, albeit one that is overshadowed by other unmeasured drivers. On the eastern slope, productivity homogeneity was jointly influenced by belowground biomass, organic matter content (0–10 cm), and total phosphorus content (10–20 cm); belowground biomass explained the largest proportion (Figure 4a). On the southern slope, influencing factors were belowground biomass and community coverage, which had independent main effects (Figure 4b). On the western slope, productivity homogeneity was jointly influenced by niche difference and alkali‐hydrolyzable nitrogen content (10–20 cm) (Figure 4c). On the northern slope, it was influenced by total potassium and total phosphorus contents (10–20 cm), with their joint effect explaining the largest proportion (Figure 4d).

**FIGURE 5:**
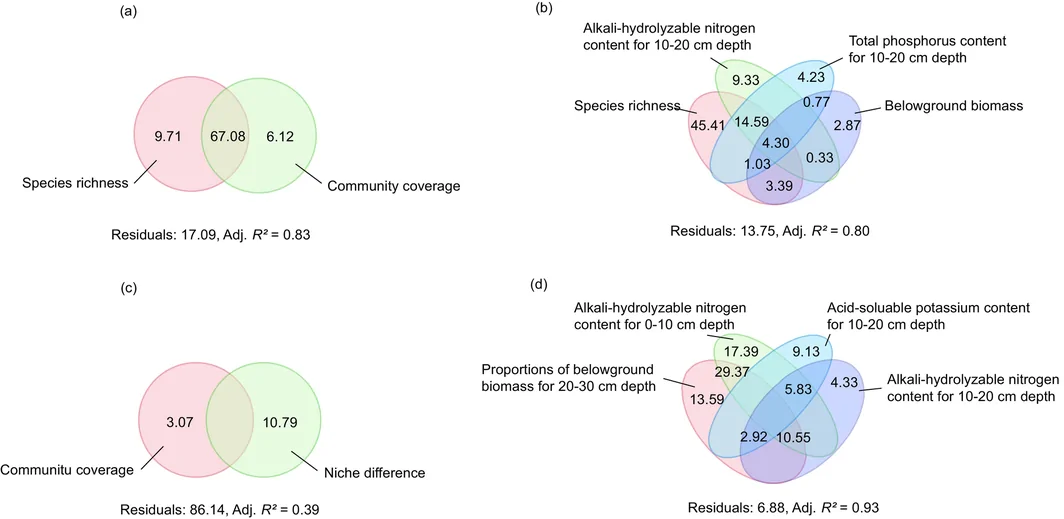
Variance partitioning analysis of species homogeneity. The numbers in the figure represent the standardized interpretation ratios (%) (Values < 0 not shown). (a) Eastern slope and (b) Southern slope and (c) Western slope and (d) Northern slope.

Similarly, among the four aspects, species homogeneity on the western slope showed a statistically significant but relatively modest association with niche difference (Figure 5), indicating that niche difference contributes to, but does not solely determine, the observed pattern. To verify whether the core conclusion is dependent on the specific calculation formula of the proxies, we recalculated niche difference using alternative indicators and repeated the main statistical analysis. The results (Figure S3) show that the proportion of niche difference explaining spatial homogeneity on the western slope is still significantly higher than that on other slopes. On the eastern slope, species homogeneity was regulated by species richness and community coverage, with their joint effect explaining the highest proportion (Figure 5a). On the southern slope, it was jointly regulated by species richness, belowground biomass, and alkali‐hydrolyzable nitrogen and total phosphorus contents (10–20 cm) (Figure 5b). On the western slope, it was regulated by community coverage and niche difference, with niche difference explaining a greater proportion but no significant interaction between them (Figure 5c). On the northern slope, it was regulated by the proportion of belowground biomass (20–30 cm) and acid‐soluble potassium (10–20 cm) and alkali‐hydrolyzable nitrogen contents (Figure 5d). A striking pattern emerged: The mechanisms regulating community homogeneity were strongly aspect‐dependent. Niche difference emerged as a significant and primary regulator of both productivity and species homogeneity exclusively on the west‐facing slope (Figures 4c and 5c). In contrast, on east‐, south‐, and north‐facing slopes, homogeneity was predominantly explained by combinations of community biomass variables and soil nutrient contents, with niche and fitness differences playing negligible direct roles.

## 4 Discussion

This study provides empirical evidence that slope aspect primarily shapes the spatial structure of alpine meadow communities, and that this effect is more closely associated with niche differences among species than with fitness differences. Three key findings emerge: First, slope aspect significantly influenced niche differences but not fitness differences. Second, variations in the spatial homogeneity of species composition across aspects aligned with the patterns of niche differences. Third, the stabilizing effect of niche difference exhibited pronounced environmental context‐dependency, becoming a key driver of community homogeneity exclusively on west‐facing slopes. We interpret these patterns through the lens of modern coexistence theory, using it as a heuristic framework to generate testable hypotheses about how aspect‐driven environmental variation might influence spatial organization.

The capacity of slope aspect to differentiate niche differences, but not fitness differences, stems from the fundamental way topography filters species by creating resource heterogeneity, rather than universally altering competitive hierarchies. Variations in solar radiation and heat reception across aspects establish distinct gradients in light, temperature, and moisture (Åström et al. 2007), which subsequently influence soil nutrient cycling and availability (Zhang, Fang, et al. 2022). This multi‐dimensional environmental heterogeneity expands the resource spectrum, facilitating differentiation in species strategies related to resource acquisition and stress tolerance (Méndez‐Toribio et al. 2016), thereby promoting niche differentiation. The niche difference index quantified in this study, integrating phylogenetic divergence and community integrity, reflects precisely this degree of strategic differentiation and species pool saturation. In contrast, fitness difference reflects the competitive hierarchy among species under specific environmental conditions (Chesson 2000). Its lack of significant variation across aspects suggests that within each aspect‐specific environment, a relatively stable competitive hierarchy—likely dominated by a few species adapted to the local conditions (e.g., *Kobresia* species)—has become established through long‐term environmental filtering (Wainwright et al. 2019). Although community structural traits (e.g., root‐to‐shoot ratio and belowground biomass) and resource conditions such as soil phosphorus content influenced both indices (Figure 3), niche difference was the index that most clearly reflected spatial heterogeneity of the environment generated by slope aspect.

The west‐facing slope experiences a combination of low water and available potassium availability coupled with high radiation stress. This multi‐dimensional stress profile, rather than a single limiting factor, may heighten the potential importance of niche differentiation as a contributing factor to spatial homogeneity. West‐facing slopes experience intense afternoon solar radiation, leading to higher surface temperatures and greater evaporative demand (Zhang, Yin, et al. 2022), thereby creating a more stressful microhabitat. This environmental filtering resulted in the lowest observed species richness and niche difference on west‐facing slopes. However, it is precisely within such resource‐limited environments where competition may intensify that any form of niche differentiation (e.g., subtle differences in water‐use timing, rooting depth, or thermal tolerance) becomes highly valuable. It can effectively reduce direct interspecies competition, thus acting as a stabilizing force that maintains community spatial structure (Ives and Carpenter 2007). The variance partitioning results of this study directly support this mechanism: Only on west‐facing slopes did niche difference show a statistically significant association with, and explained a modest fraction of, the variation in spatial homogeneity (Figures 4c and 5c).

Our finding that niche difference significantly regulated homogeneity only on the west‐facing slope is consistent with our hypothesis regarding environmental context‐dependency. This slope experiences a combination of high afternoon solar radiation and relatively low availability of water and certain potassium fractions, creating a distinct stress profile. In such a resource‐constrained environment, any existing niche differentiation may have a disproportionately important stabilizing effect, potentially reducing competitive exclusion and promoting spatial homogeneity. It may act as a partial buffer, reducing interspecies competition and thereby promoting spatial uniformity in community structure despite the overall lower species richness and niche difference values. This underscores that the absolute magnitude of niche difference may be less important than its relative role in maintaining stability under stress. In contrast, on north‐facing slopes with milder conditions, higher niche differences supported richer species pools, but homogeneity was maintained by a different suite of factors (e.g., soil nutrients, biomass), suggesting a shift from niche‐based stabilization to resource‐driven dynamics. On the resource‐impoverished east‐facing slopes, lower nutrient content may have intensified competition in a way that the existing niche differences were insufficient to exert a dominant stabilizing effect, making community structure more dependent on factors like species richness and cover. Thus, the pathway linking aspect to homogeneity is not uniform but suggests a shift toward a greater reliance on niche‐difference processes under conditions where environmental stress is greatest.

The “spatial homogeneity” framework applied here captures how persistent topographic factors shape the spatial organization of plant communities. We quantify spatial homogeneity as the inverse of variability in community biomass and species composition across local sampling points, effectively measuring the uniformity of community structure along environmental gradients (Wang and Loreau 2014). High spatial homogeneity at the slope scale indicates consistently similar community composition across space, which likely results from strong environmental filtering or widespread homogenizing dispersal. The aspect‐dependent patterns of spatial homogeneity observed in this study suggest that slope aspect acts as a persistent filter, promoting community uniformity in some contexts (e.g., on environmentally consistent north‐ or west‐facing slopes) while allowing greater spatial variability in others (e.g., on more heterogeneous east‐facing slopes). These patterns reflect long‐term species sorting and adaptation to topographic niches (Dorji et al. 2018). Future studies that link such spatial homogeneity metrics with functional trait data or environmental covariates could further clarify the ecological processes—such as niche selection, dispersal limitation, or biotic interactions—that ultimately determine how uniformly plant communities are assembled across mountain landscapes.

This study has certain limitations, but its core value lies in providing a clear, mechanism‐oriented case analysis. First, the single‐site study design limits the geographical generalizability of the conclusions. Second, the proxy indices proposed for representing fitness difference and niche difference, although grounded in theoretical logic (Chesson 2000) and showing significant correlations with community traits and soil factors (Figure 3), still require direct validation through controlled experiments or longer‐term datasets in the future. Furthermore, although we employed statistical tests robust to sample size imbalance and reported adjusted *R* ^*2*^ values, the variation in quadrat numbers across aspects remains a practical constraint in field‐based research. Nevertheless, the primary contribution of this study is the successful application of the conceptual framework of modern coexistence theory to analyze the assembly processes of natural communities in complex terrain. It clearly reveals that the slope aspect effect operates primarily through the pathway of niche difference, and the significance of this pathway increases with the intensity of environmental stress. To validate and extend this mechanism, future research should conduct replicated studies in mountain ecosystems across different regions (Liu et al. 2021) and implement long‐term monitoring to capture temporal dynamics (Ma et al. 2024). The ecological effects of slope aspect are scale‐dependent, meaning that local microtopography may be influenced by the broader topographic context. Although we adopted strict azimuth criteria, the potential influence of adjacent aspects cannot be completely ruled out. Future studies should integrate higher‐resolution topographic data and microclimatic monitoring to further resolve the multi‐scale nature of aspect effects. While our findings are consistent with the morden coexistence theory, alternative processes could also generate similar patterns. Unmeasured environmental heterogeneity (e.g., fine‐scale soil microbial composition, microtopographic variation, or local frost regimes) may covary with aspect and influence spatial homogeneity. Dispersal limitation or historical contingencies—such as past disturbance events or the order of species arrival—could shape current communities independently of contemporary niche difference. Thus, our correlational analysis cannot rule out these non‐exclusive explanations. Additionally, resource addition experiments (e.g., water, nitrogen) established along aspect gradients would provide the most direct evidence for the causal chain of “environmental heterogeneity—niche differentiation—homogeneity”.

## 5 Conclusions

In summary, by integrating modern coexistence theory with spatial homogeneity analysis, this study provides empirical evidence consistent with the hypothesis that slope aspect shapes alpine meadow plant communities, and that this effect is more closely associated with niche differences among species than with fitness differences. Furthermore, the stabilizing effect of niche difference exhibits strong environmental context‐dependency. It becomes the predominant factor driving spatial homogeneity specifically on the distinctly stressful west‐facing slope, highlighting that the operation of coexistence mechanisms is conditional on local abiotic severity. This suggests that the operation of modern coexistence mechanisms may be conditional on the local environmental context, rather than universal. Our work moves beyond simple descriptions of topographic effects, providing a testable theoretical framework and concrete case evidence for understanding how niche‐based processes underpin the spatial assembly of plant communities across mountain topographies.

## Author Contributions

**Xiaotong Gong:** data curation (lead), formal analysis (supporting), investigation (supporting), software (equal), writing – original draft (lead). **Buqing Yao:** conceptualization (lead), formal analysis (supporting), funding acquisition (supporting), methodology (supporting), software (equal), writing – original draft (supporting), writing – review and editing (supporting). **Yongxiao Li:** data curation (equal), formal analysis (equal), investigation (equal), methodology (equal), resources (supporting), validation (supporting), visualization (supporting). **Li Ma:** data curation (supporting), formal analysis (supporting), investigation (supporting). **Renqianjie Shan:** formal analysis (equal), investigation (supporting). **Ping Yang:** data curation (supporting), investigation (supporting), methodology (equal). **Huiling Zhang:** data curation (supporting), formal analysis (supporting), investigation (supporting). **Xin Li:** formal analysis (supporting), investigation (supporting). **Yaoqing Wang:** data curation (supporting), investigation (supporting). **Dengshan Zhang:** formal analysis (supporting), methodology (supporting). **Huakun Zhou:** conceptualization (supporting), methodology (supporting). **Fangping Wang:** funding acquisition (lead), methodology (lead), project administration (lead), validation (lead), writing – review and editing (lead).

## Funding

This work was supported by the study was funded by the National Nature Science Foundation of China (32160269), Natural Science Foundation of Qinghai Province of China (2025ZY037).

## Conflicts of Interest

The authors declare no conflicts of interest.

## Supporting information


**Data S1:** Supporting Information.
**Figure S1:** Contour map of the study area showing sampling locations. Red asterisks indicate the positions of sampling quadrats. Contour interval: 100 m.
**Figure S2:** Kruskal–Wallis (K–W) test for niche difference using an alternative metric of different slope aspect. Different lowercase letters indicate significant differences among habitats of different aspects according to the K–W test (*p* < 0.05).
**Figure S3:** Variance partitioning analysis of homogeneity after recalculating the niche difference. The numbers in the figure represent the standardized interpretation ratios (%) (Values < 0 not shown). (a) Productivity homogeneity. (b) Species homogeneity.
**Figure S4:** Bootstrap results for (a) productivity and (b) species homogeneity. Boxplots show the distribution of mean homogeneity across 500 resampled baselines; black dots indicate original baseline values. Original values lie within the 95% confidence intervals for all aspects, demonstrating robustness to baseline selection.
**Table S1:** Kruskal–Wallis (K–W) tests for the community characteristics of the different slope aspects.

## Data Availability

The data that support the findings of this study are openly available in figshare at https://doi.org/10.6084/m9.figshare.30899729, reference number 30899729.
