# Supplementary material for: Slope Aspect Modulates Alpine Meadow Spatial Homogeneity Through Niche Differences, Not Fitness Differences
Source: Ecol Evol. 2026 Sep 1;16(9):e74255. doi: 10.1002/ece3.74255 (PMC13531361; doi:10.1002/ece3.74255)
Supplement: Supplementary file 1 [file ECE3-16-e74255-s001.zip › ece374255-sup-0005-TableS1.docx]

**Table S1:** Kruskal–Wallis (K-W) tests for the community characteristics of the different slope aspects.

| Index | Eastern slope | Southern slope | Western slope | Northern slope |
| --- | --- | --- | --- | --- |
| Species richness | 15.47±8.17^ab^ | 13.67±8.58^ab^ | 14.30±5.58^b^ | 14.46±9.12^a^ |
| Community height | 13.77±5.90^a^ | 14.08±7.03^a^ | 10.73±2.01^a^ | 11.93±4.07^a^ |
| Community coverage | 0.91±0.16^a^ | 0.88±0.19^ab^ | 0.86±0.09^b^ | 0.91±0.10^ab^ |
| Aboveground biomass | 56.24±25.59^ab^ | 66.16±34.37^a^ | 38.70±14.18^b^ | 53.08±16.44^ab^ |
| Proportions of belowground biomass for the 0–10 cm deep soil layer | 0.88±0.07^b^ | 0.84±0.12^b^ | 0.93±0.02^a^ | 0.89±0.06^ab^ |
| Proportions of belowground biomass for the 10–20 cm deep soil layer | 0.10±0.07^a^ | 0.13±0.11^a^ | 0.06±0.01^b^ | 0.09±0.06^a^ |
| Proportions of belowground biomass for the 20–30 cm deep soil layer | 0.03±0.03^ab^ | 0.04±0.03^a^ | 0.02±0.01^b^ | 0.02±0.01^ab^ |
| Belowground biomass | 1,034.94±551.87^bc^ | 854.75±510.32^c^ | 1,423.03±429.77^a^ | 1,097.53±409.65^ab^ |
| Root-to-shoot ratio | 23.37±19.42^bc^ | 15.37±11.41^c^ | 38.09±14.48^a^ | 25.02±15.02^ab^ |

Notes: The values in the table are the mean ± SD, and the different lowercase letters indicate significant differences among the habitats of different aspects (*P* < 0.05).
