# Supplementary material for: Slope Aspect Modulates Alpine Meadow Spatial Homogeneity Through Niche Differences, Not Fitness Differences
Source: Ecol Evol. 2026 Sep 1;16(9):e74255. doi: 10.1002/ece3.74255 (PMC13531361; doi:10.1002/ece3.74255)
Supplement: Supplementary file 1 [file ECE3-16-e74255-s001.zip › ece374255-sup-0006-Supinfo.docx]

**Slope Aspect Modulates Alpine Meadow Spatial Homogeneity through Niche Differences, Not Fitness Differences**

**Sensitivity analysis: The fitness and niche difference on the robustness of different proxies**

To verify whether the core conclusion is dependent on the specific calculation formula of the proxies, we recalculated fitness difference and niche difference using alternative indicators and repeated the main statistical analysis. For fitness difference, we used the Heip index instead of the Pielou evenness index; for niche difference, we calculated the standardized value based on the average pairwise phylogenetic distance (MPD), multiplying community integrity by MPD. The results (Fig. S2, S3) show that the key conclusions have not undergone substantial changes: niche difference still shows the highest value on the northern slope and the lowest on the western slope (*P* < 0.05), and the proportion of niche difference explaining spatial homogeneity on the western slope is still significantly higher than that on other slopes. Therefore, our main findings are not the product of a specific mathematical form and have good robustness.

**Bootstrap sensitivity analysis: Productivity homogeneity and species homogeneity on the robustness of baseline quadrat selection**

To test whether the core indicators (species homogeneity and productivity homogeneity) depend on the selection of specific baseline quadrats within each slope direction, we conducted sensitivity analyses using the Bootstrap resampling method. The results show that the productivity homogeneity for each slope aspect all fall within the corresponding 95% confidence interval of the Bootstrap (Figure. S4(a)). This indicates that the productivity homogeneity is not dependent on a specific baseline quadrat. Similarly, the species homogeneity for all slope aspects all fall within the 95% confidence interval of their respective Bootstrap distributions (Figure. S4(b)). This suggests that the species homogeneity is also not sensitive to the choice of the baseline quadrat. The sensitivity analysis above verifies that the productivity homogeneity and species homogeneity used in this study have good mathematical robustness, and the core pattern is not driven by a single baseline plot. Therefore, the subsequent statistical inferences based on these indicators are reliable.
